# Eigenvector Centrality Mapping for Analyzing Connectivity Patterns in fMRI Data of the Human Brain

**DOI:** 10.1371/journal.pone.0010232

**Published:** 2010-04-27

**Authors:** Gabriele Lohmann, Daniel S. Margulies, Annette Horstmann, Burkhard Pleger, Joeran Lepsien, Dirk Goldhahn, Haiko Schloegl, Michael Stumvoll, Arno Villringer, Robert Turner

**Affiliations:** 1 Max Planck Institute for Human Cognitive and Brain Sciences, Leipzig, Germany; 2 Department of Medicine, University of Leipzig, Leipzig, Germany; Indiana University, United States of America

## Abstract

Functional magnetic resonance data acquired in a task-absent condition (“resting state”) require new data analysis techniques that do not depend on an activation model. In this work, we introduce an alternative assumption- and parameter-free method based on a particular form of node centrality called eigenvector centrality. Eigenvector centrality attributes a value to each voxel in the brain such that a voxel receives a large value if it is strongly correlated with many other nodes that are themselves central within the network. Google's PageRank algorithm is a variant of eigenvector centrality. Thus far, other centrality measures - in particular “betweenness centrality” - have been applied to fMRI data using a pre-selected set of nodes consisting of several hundred elements. Eigenvector centrality is computationally much more efficient than betweenness centrality and does not require thresholding of similarity values so that it can be applied to thousands of voxels in a region of interest covering the entire cerebrum which would have been infeasible using betweenness centrality. Eigenvector centrality can be used on a variety of different similarity metrics. Here, we present applications based on linear correlations and on spectral coherences between fMRI times series. This latter approach allows us to draw conclusions of connectivity patterns in different spectral bands. We apply this method to fMRI data in task-absent conditions where subjects were in states of hunger or satiety. We show that eigenvector centrality is modulated by the state that the subjects were in. Our analyses demonstrate that eigenvector centrality is a computationally efficient tool for capturing intrinsic neural architecture on a voxel-wise level.

## Introduction

Functional magnetic resonance data (fMRI) of the human brain acquired in a task-absent (“resting state”) condition has attracted increasing interest in recent years. Due to the absence of an experimental paradigm, analysis procedures based on an activation model are not applicable. New types of techniques have been developed focusing on functional connectivity rather than task activation. For instance, correlation of time series between a pre-specified seed region and all other voxels of the brain is robust and conceptually clear. However, it can only be successfully applied if some prior knowledge exists for identifying seed regions. Another widely used technique is based on independent component analysis (ICA) whose primary advantage is its freedom from hypotheses preceding the analysis and the need for selecting seed regions [1]. However, the number of independent components is difficult to specify and assumptions must be made about what constitutes a valid network. For comprehensive reviews of the above and related methods see [2], [3].

More recently however, graph-based methods have been proposed for the analysis of functional and structural magnetic resonance data of the human brain. Their main feature is that they take brain regions as nodes in a graph. Some of these methods have also been applied to the analysis of resting state fMRI data [4]–[6]. Given the small world properties of the human brain [7], [8], graph-based methods provide a valuable tool for elucidating network structures.

In the present study, we focus on a particular type of graph-based method that identifies nodes which play central roles within the network structure. Such nodes are characterized by a measure called “node centrality”. Node centrality is a key concept in social network analysis of which several competing definitions exist and some of which have been applied to fMRI data analysis in the past [5], [9]. Here we discuss several of these approaches - in particular “betweenness centrality”, “degree centrality” and “eigenvector centrality”. Sporns et al. [9] for instance advocate a combination of various graph measures including degree, betweenness centrality and closeness centrality.

Thus far, centrality measures have been applied to a pre-selected set of nodes consisting of at most several hundred elements (e.g. [6], [8], [9]). Here, we propose to apply this measure to all voxels in a region of interest covering the entire cerebrum thereby avoiding any selection bias [10].

However, due to computational complexity, closeness and betweenness centrality measures are not suited for compiling brain maps with thousands of voxels. Therefore in this study, we will focus primarily on ‘eigenvector centrality’ [11], [12]. To our knowledge, eigenvector centrality has not yet been used in the context of fMRI data analysis. Eigenvector centrality specifically weights nodes based on their degree of connection within the network. It does so by counting both the number and the quality of connections so that a node with few connections to some high-ranking other nodes may outrank one with a larger number of mediocre contacts [13]. Google's “PageRank” algorithm is a variant of eigenvector centrality [14]. Both the human brain and the world wide web exhibit small world properties suggesting that an algorithm that is effective as part of a search engine may also be effective in analyzing network properties of the human brain.

Eigenvector centrality can be used on a variety of different similarity metrics. Here, we present applications based on linear correlations and on spectral coherences between times series. This latter approach allows us to draw conclusions about connectivity patterns in different spectral bands. The motivation for choosing spectral measures came from Salvador et al. [15] who have emphasized the importance of investigating interregional dependencies in the frequency domain rather than in the time domain.

We propose to use eigenvector centrality as a mapping tool for the entire brain or parts of it. Such maps can be subjected to statistical tests to detect groupwise differences in centrality between experimental states. For abbreviation, we will call this method ECM (Eigenvector Centrality Mapping).

## Materials and Methods

Several definitions of node centrality exist - each having a slightly different interpretation. Common to all of these definitions is that they are based on a symmetric matrix containing pairwise similarity measures. Let 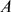 be such an 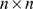 similarity matrix where entries 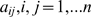 contain a pairwise similarity measure between time series in voxels 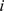 and 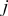. The number of voxels 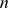 is determined by user-specified regions of interest (ROI) to which all subsequent analysis steps are restricted. In the experiments reported in this study, the ROI covered the entire brain excluding the cerebellum and consisted of 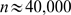 voxels.

The matrix 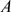 is symmetric so that each voxel can be viewed as a node in an undirected weighted graph in which similarity values correspond to weights along the edges of the graph. In graph-based applications, these weights represent distances between nodes and are therefore non-negative. As a result, centrality measures are generally also defined to be non-negative. See Bonacich [16] for a discussion of this point.

### Degree centrality

The simplest centrality measure is called “degree centrality”. The degree 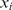 of a node 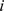 is defined as
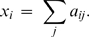
Thus, a node has a high degree if it has strong connections to many other nodes in the graph.

### Eigenvector centrality

Eigenvector centrality was first introduced by Bonacich [11], [12] and a later variant of it is a central part of Google's PageRank algorithm [14]. Much like degree centrality, it favours nodes that have high correlations with many other nodes. However, in contrast to degree centrality it specifically favours nodes that are connected to nodes that are themselves central within the network. Thus it takes into account the entire pattern of the network.

As before let 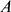 denote an 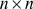 similarity matrix. Then the eigenvector centrality 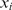 of node 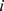 is defined as the 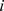-th entry in the normalized eigenvector belonging to the largest eigenvalue of 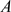. Note that with this definition 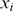 fulfils the characteristics described above. To see why let 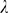 be the largest eigenvalue and 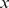 the corresponding eigenvector, then
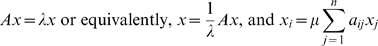
with proportionality factor 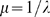 so that 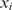 is proportional to the sum of similarity scores of all nodes connected to it.

Uniqueness of this definition is ensured by the Perron-Frobenius theorem which states that any square matrix with strictly positive entries has a unique largest real eigenvalue with strictly positive components. This is also true for irreducible square matrices with non-negative entries. An irreducible matrix has at least one non-zero off-diagonal element in each row and column.

Since we assume that 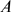 represents distances between nodes we have 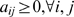. In the present context, we may assume that 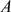 is irreducible because fMRI time series are almost never entirely dissimilar so that a sufficient number of non-zero entries in 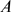 exist. Thus, an eigenvector belonging to the normalized largest eigenvalue exists and its entries 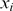 provide a centrality measure for each node 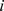 which is uniquely defined and non-negative. Note that symmetric matrices with negative entries may have several largest eigenvalues that are not distinct so that the requirement of non-negativity is essential for ensuring the uniqueness of this definition (see Appendix S1 for an example).

Eigenvector centrality is related to principal components analysis (PCA) in that both methods are based on eigenvector decompositions of similarity matrices. However, PCA differs from eigenvector centrality in that it only allows linear correlations as a similarity metric. But linear correlations may be negative so that the first principal component is not uniquely defined because of possible multiplicities of eigenvalues.

In our experiments we used linear correlations which were re-scaled to be non-negative and also a spectral coherence metric which is non-negative by definition (see below). Other similarity metrics such as mutual information or wavelet transform coherence (WTC) [17] might be used for eigenvector centrality mapping (ECM) as well.

Many algorithms for computing eigenvectors of symmetric matrices are known. In the present context, it suffices to find the eigenvector belonging to the largest eigenvalue. For this special case, the power iteration method [18, 405ff] is one of the most efficient, and was used in our experiments.

### Betweenness centrality

The betweenness centrality 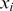 of some node 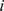 is defined as:
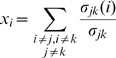
where 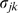 is the number of shortest geodesic paths from 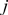 to 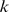, and 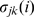 is the number of shortest geodesic paths from 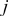 to 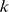 that pass through node 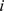. This is normalized by dividing through the number of pairs of nodes not including 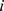, which is 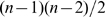.

Betweennnesss centrality is computationally expensive. For weighted graphs, its complexity is 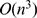 which can be reduced for unweighted graphs to 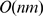 where 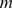 is the number of edges (non-zero correlations) [19] making it computationally impracticable for large values of 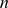 or 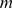. Note that generally, correlations are thresholded at some user-defined level prior to applying betweenness centrality. It was used e.g. by He et al. [6] for analyzing spontaneous fluctuations in a network consisting of 90 regions of interest. We tested betweenness centrality on a region of interest containing 17,398 voxels that covered parts of the left hemisphere of one subject. The computation took 26 hours using 4 parallel 2.6 GHz processors for a single data set. Application to a region of interest with full brain coverage was not feasible.

### Linear correlation

Linear correlation has been proposed as a metric for analysing functional connectivity [20]. A high positive correlation between two fMRI time series indicates a strong similarity, a high negative a strong dissimilarity. Note that this measure is quite agnostic about any form of causal influence between brain regions. It is defined as follows.

Let 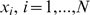 and 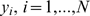 be time series of length 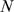 in two voxels 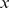 and 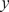. Their correlation is defined as
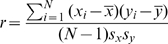
where 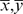 denote the sample mean and 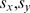 the standard deviations.

Because the similarity matrix 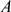 should be positive, linear correlations between time series must be re-scaled accordingly. We propose to use
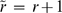
where 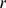 denotes the correlation between two time series and 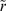 the corresponding scaled version. Note however that strong negative correlations may indicate some form of inverse coupling. Therefore, an alternative way to handle negative correlations might be to take absolute values instead of the approach proposed here.

### Spectral coherence

Salvador et al. [15] have noted that interregional dependencies can be more readily observed in the frequency domain than in the time domain. Therefore, we have also used frequency based similarity metrics for ECM. Specifically, we employ spectral coherence for this purpose. It has been previously applied to fMRI data analysis [15], [21], [22]. In the following, we give a brief overview. For more information see for instance [23], [24].

Let 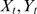 denote real-valued stationary time series in two voxels, and let 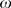 be some frequency of interest. We assume that they are normalized to zero mean. Their cross-correlation function evaluated at lag 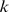 is defined as
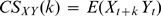
and the corresponding cross-spectral density is:
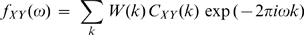
Analogously, the auto-spectral density of a single time course 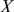 is
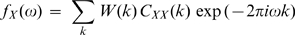
Several choices for the weighting factors 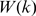 exist. Among the most common ones are Parzen or Tukey windows. Here, we used the Tukey window which is defined as:
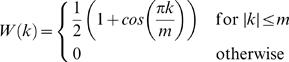
where 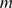 is the number of lags to compute the autocorrelation for. As a rule of thumb, 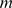 should be chosen to be in the range 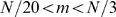 where 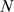 is the length of the time series [25, p.141]. In the data presented below the time series length was 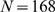, and we used 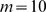 throughout.

Since 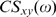 is not necessarily symmetric the cross-spectrum is generally a complex function. The real part of 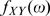 is known as the cospectrum denoted as 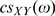 and the imaginary part as the quadrature spectrum 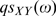. The spectral coherence 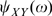 between 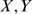 at frequency 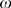 is defined as:
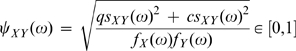



Note that information about phase lags is not included in the above measure. Frequency-dependent phase coherence can be computed using the above definitions as follows
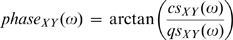



### Experiment 1

Functional MRI/EPI data were acquired of 35 normal volunteers on a 3T MRI scanner (Siemens Tim Trio) using TR = 2.3 sec, TE = 30ms, 3×3 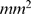 in-plane resolution, 3 mm slice thickness, 1 mm gap between slices. Each scanning session began with a task-absent (“resting state”) scan lasting 7.6 minutes during which subjects were asked to fixate a fixation cross. A second resting state scan with the same acquisition parameters followed about 10 minutes later within the same scanning session. In between these two scans, subjects were scanned in another task absent condition using sagittal instead of axial slices. Data from this scan were not used for the present study.

**Figure 1:**
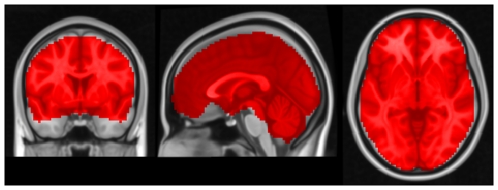
The region of interest in experiment 1. The mask covering the entire brain including the cerebellum containing about 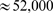 voxels. MNI coordinates of slice positions are (0,0,0).

All data sets were initially fieldmap corrected using the software system Lipsia [26]. Data preprocessing then continued using FSL [27], and consisted of motion correction, bandpass filtering (SPECS), and spatial smoothing (SPECS). Finally, preprocessed data sets were registered into standard MNI152 (Montreal Neurological Institute) brain space using FSL's nonlinear registration software FNIRT, and resampled to an isotropic voxel grid with a resolution of 3×3×3 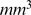. We manually defined a region of interest containing about 52,000 voxels covering the entire cerebrum to which subsequent ECM analysis was applied (Figure 1).

### Experiment 2

Functional MRI/EPI data were acquired of 22 normal volunteers on a 3T MRI scanner (Siemens Tim Trio) using TR = 2.3 sec, TE = 30ms, 3×3 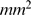 in-plane resolution, 3 mm slice thickness, 1 mm gap between slices. The study was approved by the ethics committee of the University of Leipzig. All subjects have written informed consent. The subjects were asked to attend two scanning sessions, in one of which they were asked to refrain from eating after 6 pm of the previous day. During both sessions, we first acquired resting state data for 6.5 minutes during which subjects were asked to fixate a fixation cross. During the following 34 minutes they were shown pictures of food and tools that they were asked to respond to by button presses. Finally, another 6.5 minutes of resting state data were acquired. In the present study, we only analyzed the initial resting state data acquired before visual stimulation began, and ignored the rest of the experiment.

**Figure 2:**
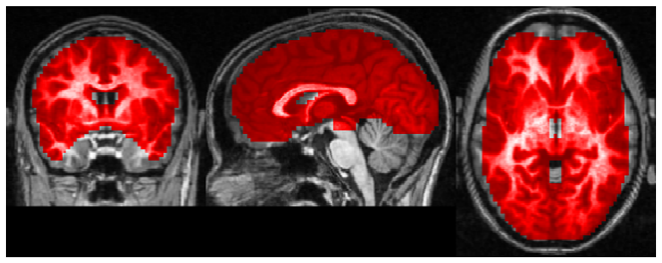
The region of interest in experiment 2. The mask used in experiment 2 containing about 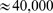 voxels. Talairach coordinates of slice positions are (0,0,0).

Data processing was done using the software system Lipsia [26]. All data sets were initially corrected for motion and slicetime offsets. A baseline correction was applied using a highpass filter with a cutoff frequency of 1/90 Hz, and a spatial smoothing with a Gaussian filter of fwhm = 8 mm was used. All data sets were initially registered to an AC/PC coordinate system where the data were resampled to an isotropic voxel grid with a resolution of 3×3×3 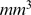. We manually defined a mask containing 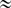 40,000 voxels covering the entire brain while excluding the cerebellum and parts of CSF (Figure 2).

### Data processing

For both experiments, we computed pairwise similarity matrices between time series of any two voxels inside the mask using scaled linear correlation and for experiment 2 also spectral coherence, and applied the ECM algorithm to these matrices. The resulting centrality maps were then transformed as described by van Albada et al. [28] in order to ensure that they obey a Gaussian normal distribution as required for subsequent statistical tests. The results were corrected for multiple comparisons using cluster-size and cluster-value thresholds obtained by Monte-Carlo simulations [29], [30] using a significance level of 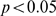. Clusters in the resulting maps were obtained using an initial z-value threshold of 2.33. The Monte Carlo simulation determines the size and peak value a cluster must have in order to be considered statistically significant. Thus, a cluster with only a moderately high peak value might be considered significant if it is large enough. On the other hand, a cluster with a very high peak value might be significant even if it is rather small. Computation times for ECM were about 20 minutes per dataset on a 2.6 GHz Opteron processor. About 6 GByte of computer memory are needed to store an 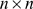 matrix with 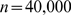 voxels to cover the cerebrum at 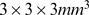 resolution.

## Results

### Experiment 1

**Figure 3:**
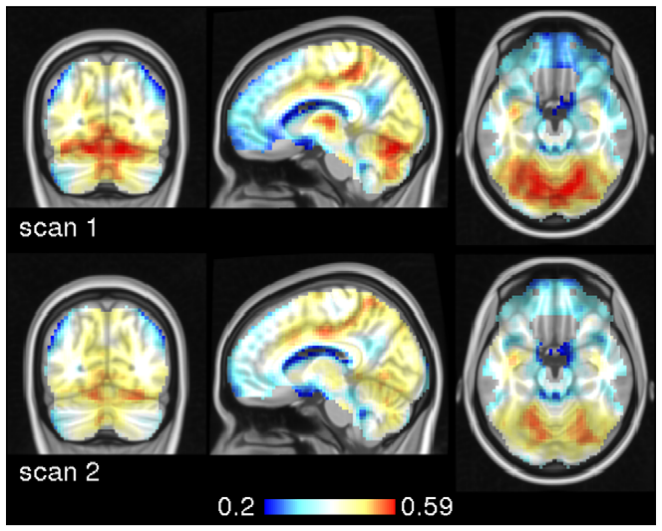
Group averages of eigenvector centrality maps in experiment 1. The group average of the first scan is shown in the top row. The bottom row shows results of the second scan. The similarity metric was scaled linear correlation. MNI coordinates of slice positions are (−4,−71,−20).

**Figure 4:**
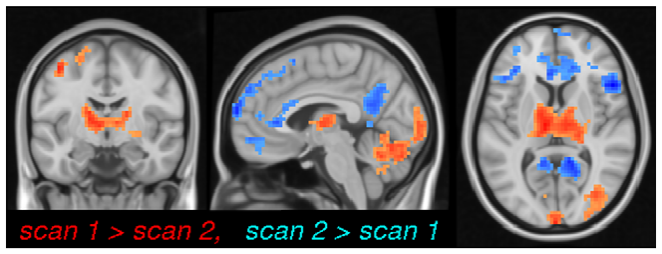
Pairwise t-test between the two ECMs of experiment 1. Results are thresholded at 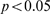 (corrected). MNI coordinates of slice positions are (−4,−8,7).

Figure 3 shows group averages of eigenvector centrality in the two scans. A network of hubs including sensorimotor areas of the marginal ramus of the cingulate and mid-cingulate, thalamus, primary visual cortex, insula and operculum are common to both. Figure 4 shows results of a paired t-test contrasting the two scans. During the first scan, eigenvector centrality scores were significantly higher in left and right thalamus and in the cerebellum. During the second scan, eigenvector centrality was larger in posterior cingulate, medial frontal and right opercular cortices, and medial frontal areas.

**Figure 5:**
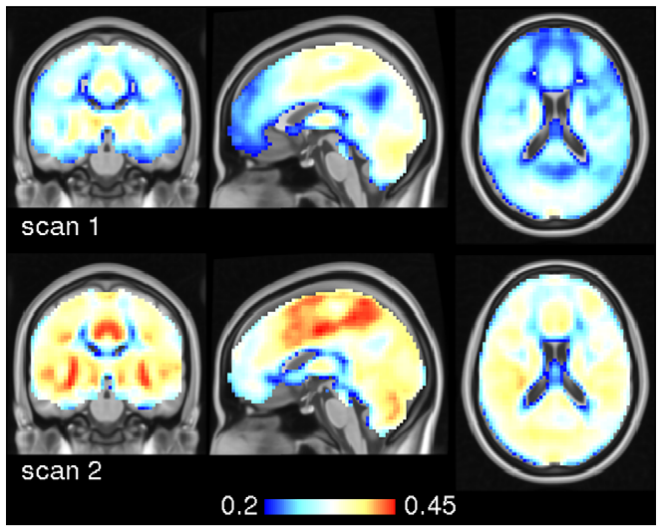
Group averages of degree centrality maps in experiment 1. The similarity metric was scaled linear correlation. Note that degree centrality is larger almost everywhere in the brain during the second scan. MNI coordinates of slice positions are (0,−17,18).

For comparison, we additionally computed another centrality map - this time using degree centrality instead of eigenvector centrality (Figure 5). Note that during the second scan, degree centrality was larger almost everywhere in the brain.

### Experiment 2

**Figure 6:**
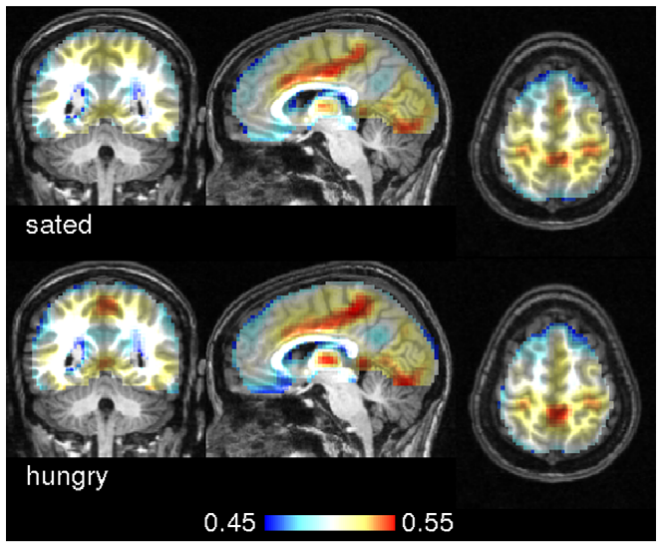
Group averages of eigenvector centrality maps in experiment 2. The top row shows group averages of eigenvector centrality maps of subjects in the sated state. Below, the group average across the hungry state is shown. The similarity metric used here was scaled linear correlation. Talairach coordinates of slice positions are (4,−49,58).

**Figure 7:**
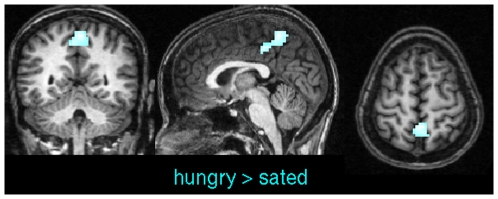
Pairwise t-test between sated and hungry subjects using scaled linear correlations in experiment 2. Results are thresholded at 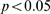 (corrected). Centrality values in precuneus were significantly higher during the hungry state. Other regions did not show significant effects. Talairach coordinates of slice positions are (−2,−50,56).

Figure 6 shows group averages of ECM based on scaled linear correlation. The overall ECM pattern looked very similar although we found statistically significant differences in precuneus when contrasting hungry against sated state (Figure 7 and Appendix S2). Recent literature postulated that posterior midline cortex, comprising precuneus and posterior cingulate cortex constitutes the core hub within the default network of the human brain with strong connections to ventral medial prefrontal cortex/anterior cingulate cortex, inferior lateral parietal cortex, and the hippocampi [31], [32]. This particular portion of the precuneus, located in the anterior section adjacent to the marginal ramus of the cingulate sulcus, has been implicated in self-related processing (in contrast to the episodic memory-related role of the posterior precuneus) [33]. Although the enhanced centrality of precuneus during the hungry state indicates a changed core hub of the default network, we found no other areas with significantly changed centrality values across the brain. Nonetheless, the increased centrality of anterior precuneus during the hungry state is consistent with the proposed self-related functionality of this region.

**Figure 8:**
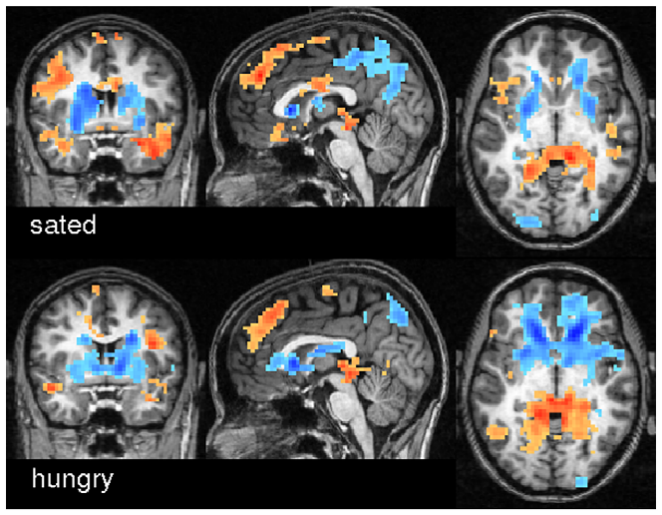
Variations in eigenvector centrality across frequency bands in experiment 2. The maps show a t-test contrasting spectral ECMs of 1/10, 1/15, 1/20 Hz versus 1/25, 1/20, 1/35 Hz. Blue colors indicate regions were higher frequencies showed stronger centrality. Red colors indicate regions where very low frequencies dominate. The maps are thresholded at 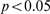 (corrected). Talairach coordinates of slice positions are (0,0,0).

We next employed spectral coherence to investigate frequency based similarity metrics because of their known advantages in the observation of interregional dependencies [15]. We found strong effects of frequency in ECM across spectral bands as shown in Figure 8. In frequency bands 1/10 Hz up to 1/20 Hz, ECM was significantly larger in precuneus, the striatum and several more areas. Very low frequency bands (1/25 Hz, 1/30 Hz, 1/35 Hz) dominate at the temporal poles and mediodorsal frontal areas.

**Figure 9:**
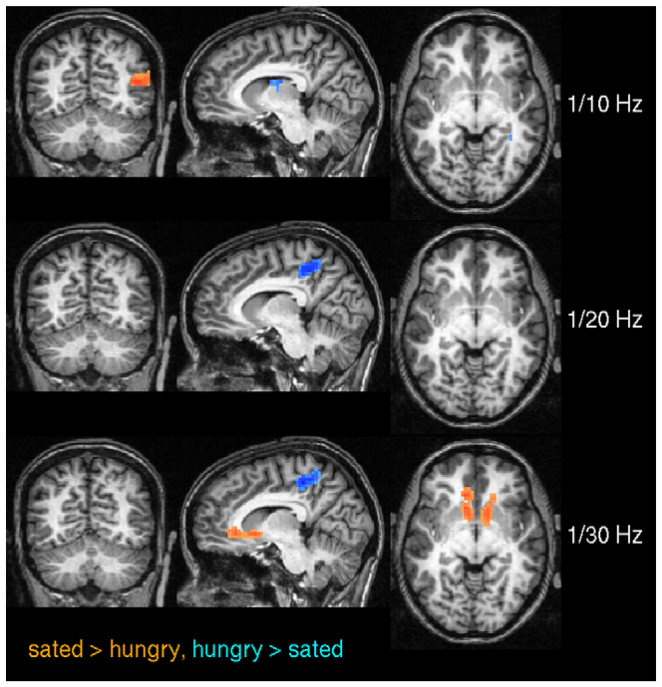
Pairwise t-test between sated and hungry subjects using spectral coherence in experiment 2. The results are shown for three frequencies (0.1 Hz, 0.05 Hz, 0.033 Hz) thresholded at p 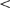 0.05 (corrected). Voxels where centrality values were significantly larger in the sated state are shown in red, the reverse is shown in blue. Note that the difference in precuneus is only present at frequencies 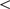 0.1 Hz. At 0.033 Hz a significant difference becomes apparent at the ventral striatum. Talairach coordinates of slice positions are (−8,−60,−2), see Table 1.

Figure 9 and Table 1 show differences between the sated and the hungry state across various spectral bands based on spectral coherence. In particular, differences appear in the anterior precuneus at 1/20 Hz and 1/30 Hz, and in the ventral striatum at 1/30 Hz.

**Table 1:** Significant differences between hungry and sated state using spectral coherence in experiment 2.

frequency		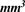	coordinates
1/10 Hz	sup. front. sulc.	3618	(26, −7, 48)
	intrapariet. sulc.	3186	(35, −61, 18)
	thalamus	3429	(8, −7, 21)
1/20 Hz	precuneus	7857	(8, −46, 48)
	sup. temp. sulc.	324	(56, −19, −12)
1/30 Hz	ventral striatum	4725	(−10, 26, 3)
	precuneus	5589	(−4, −40, 51)

List of regions showing a significant difference in ECM between hungry and sated state using spectral coherence at 1/10, 1/20, 1/30 Hz (see Figure 9). The results are corrected for multiple comparison at 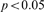. Only regions larger than 100 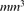 are listed. Coordinates of the peak voxel are given in the Talairach system.

## Discussion

We propose eigenvector centrality as a new method for analyzing fMRI data. It is parameter-free, computationally fast and does not depend on prior assumptions. In contrast to previous studies using centrality measures [6], [8], [9], we have applied them here to a large region of interest consisting of thousands of voxels. Under those circumstances, betweenness centrality becomes computationally intractable. The computational speed allowed us to obtain whole brain centrality maps and use them in a manner similar to contrast maps obtained in standard regression analyses.

In the first experiment, we found significant differences between ECMs of two resting state scans following each other within the same session. In particular, left and right thalamus had higher eigenvector centrality scores during the first scan. Thalamus has been implicated in mediating attention and arousal in humans [34], [35] suggesting that subjects' attention and/or arousal may have declined with time spent in the scanner. We also found higher centrality in the cerebellum during the first scan. The cerebellum is involved in the coordination of voluntary motor movement and muscle tone. Perhaps the mental effort of remaining motionless for a prolonged period of time may have played a role in this context [36].

On the other hand, posterior cingulate and anterior medial frontal cortex appeared stronger in the second scan - regions that are associated with the “default mode network” [37]. A possible explanation might be that subjects were more relaxed and more “at rest” during the second scan so that the typical “default mode” pattern emerged more clearly.

For comparison, we also computed degree centrality and found that during a second resting state scan, degree centrality increased almost everywhere indicating a general increase in correlations across the brain. This may be due to a global physiological influence such as respiration or heart rate. Eigenvector centrality on the other hand did not show such a global effect. Rather it highlighted specific regions that were differentially affected by the prolonged duration of the experiment.

For the second experiment, we additionally used frequency instead of time based similarity metrics with the known advantages in the detection of interregional dependencies [15], we identified regions with significant changes in their centrality scores that match well with previous findings from experiments addressing paradigms related to food and eating in hungry and sated state [38]–[40]. We found the ventral striatum as the most prominent region within the network (in the 1/30 Hz band, see Figure 9) which is well known as a key region implicated in reward, e.g. [41] such as consummatory food [42], and displays functional connectivity throughout the prefrontal and motor cortex [43].

The spectral coherence measure assumes that the coupling between fMRI time series is stationary over time. This assumption may sometimes be unrealistic. In such cases, the wavelet transform coherence (WTC) [17] might be better suited because it describes coherence and phase lag between two time series as a function of both time and frequency. It has recently been used for analyzing resting state fMRI data [44].

For the present work, we have only used spectral coherence but not phase coherence. However, it might be advantageous to include phase coherence and use it in conjunction with spectral coherence. We plan to explore that possibility in future work.

In both experiments, we found high centrality values in cortical and subcortical areas, but also in white matter regions. This agrees with results found by Mezer et al. [45] who reported clusters of similar BOLD fluctuations not only in the cortical and subcortical regions, but also within the white matter. The origin of such effects is still unclear and remains the object of future research.

It should be noted that low frequency fluctuations may also be caused by aliasing effects (undersampling) so that the actual sources of these signals need not be in that same low frequency range. Nonetheless, recent studies have confirmed that oscillations - even at very low frequencies - appear robust and reliable [46], [47] so that these results are not unexpected. It remains to be shown whether these findings indicate the existence of natural frequencies at which specific networks operate. Such natural frequencies have recently been postulated by Rosanova et al. [48] for the human corticothalamic circuits based on EEG and TMS data. Our findings suggest that analogous patterns might exist at much lower frequencies observable in fMRI even though the exact nature of these connectivity patterns remains to be investigated. In this context, it may be interesting to use alternative frequency-dependent similarity metrics as described e.g. in Salvador et al. [15].

The initial analyses presented in this study demonstrate that eigenvector centrality is a computationally efficient tool for capturing intrinsic neural architecture on a voxel-wise level. The independence of centrality approaches from a priori hypotheses, makes it a valuable methodological addition to the “model-free” analytic toolbox.

## Supporting Information

Appendix S1 A symmetric matrix with non-unique eigenvalues.
(0.02 MB PDF)

Appendix S2 Axial slices of ECM group averages in experiment 2.
(0.34 MB PDF)
