# Supplementary material for: Eigenvector Centrality Mapping for Analyzing Connectivity Patterns in fMRI Data of the Human Brain
Source: PLoS One. 2010 Apr 27;5(4):e10232. doi: 10.1371/journal.pone.0010232 (PMC2860504; doi:10.1371/journal.pone.0010232)
Supplement: Appendix S1 — A symmetric matrix with non-unique eigenvalues. (0.02 MB PDF) [file pone.0010232.s001.pdf]

Supporting Information 1

Example of a symmetric matrix A with negative values and multiple largest eigenvalues so that the first principal component is not unique. Note that A is positive semidefinite and symmetric (as are covariance matrices). The columns in matrix E are the eigenvectors of A with eigenvalues $\lambda_1 = 1.2, \;\; \lambda_2 = 1.2, \;\; \lambda_3 = 0.6$.

$$\mathrm{A} \; = \begin{pmatrix} 1.000 & 0.200 & 0.200 \\ 0.200 & 1.000 & -0.200 \\ 0.200 & -0.200 & 1.000 \end{pmatrix}, \;\; \mathrm{E} \; = \begin{pmatrix} 0.816 & 0.000 & 0.577 \\ 0.408 & -0.707 & -0.577 \\ 0.408 & 0.707 & -0.577 \end{pmatrix}$$
