# Supplementary figures and images for: Eigenvector Centrality Mapping for Analyzing Connectivity Patterns in fMRI Data of the Human Brain
Source: PLoS One. 2010 Apr 27;5(4):e10232. doi: 10.1371/journal.pone.0010232 (PMC2860504; doi:10.1371/journal.pone.0010232)

## Supporting Information 2

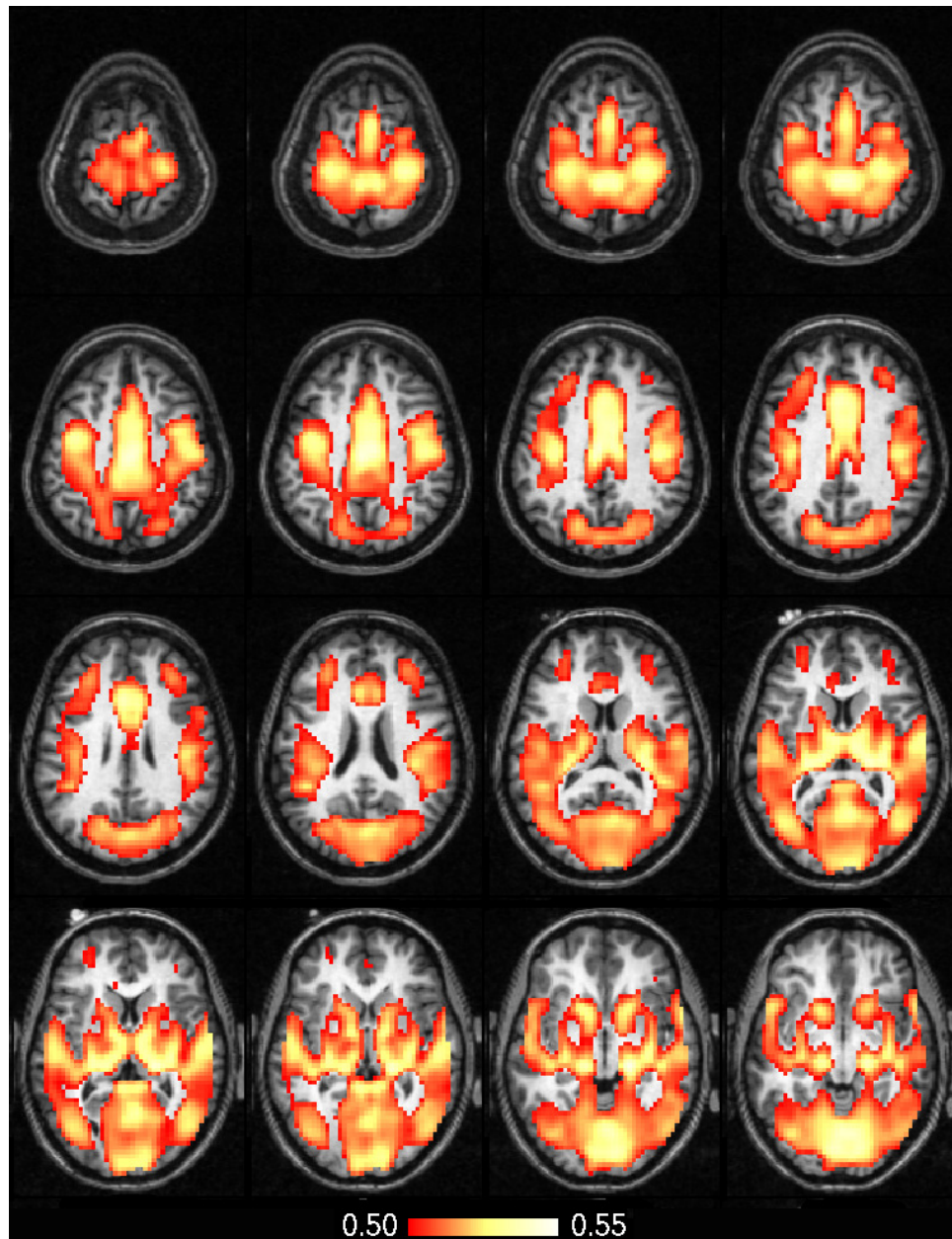

Axial slices of ECM group averages in experiment 2 (sated state).

Supplement: Appendix S2 — Axial slices of ECM group averages in experiment 2. (0.34 MB PDF) [file pone.0010232.s002.pdf]
